# Mediator and TREX-2: Emerging links between transcription initiation and mRNA export

**DOI:** 10.1080/19491034.2016.1169352

**Published:** 2016-03-30

**Authors:** Tobias Schubert, Alwin Köhler

**Affiliations:** Max F. Perutz Laboratories, Medical University of Vienna, Vienna Biocenter Campus (VBC), Vienna, Austria

**Keywords:** mediator, nuclear pore complex, transcription coupled mRNA export;, TREX-2

## Abstract

Nuclear pore proteins interact dynamically with chromatin to regulate gene activities. A key question is how nucleoporin interactions mechanistically alter a gene's intranuclear position and transcriptional output. We reported recently on a direct interaction between the nuclear pore-associated TREX-2 complex and promoter-bound Mediator. This highlights how nuclear-pore associated adaptors gain regulatory access to the core transcription machinery. In this Extra View, we discuss an additional implication that arises from our work and the recent literature: how promoter elements may regulate mRNA metabolism beyond transcription initiation.

## Introduction

**Figure 1.**
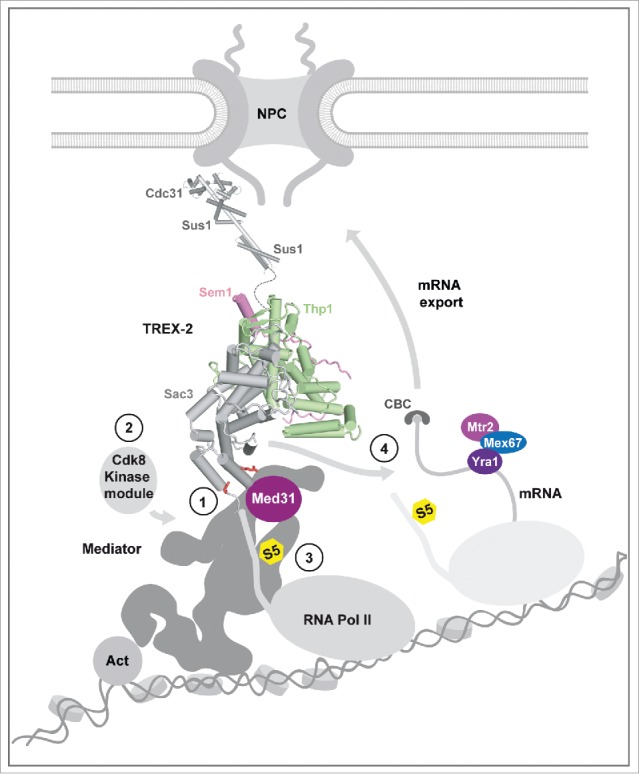
Mechanism for a relay between TREX-2, Mediator, and Pol II. Model depicts the putative overall topology of the NPC-associated TREX-2 complex and its interaction with Mediator. Mediator cartoon follows the outline of the yeast Mediator cryo-EM structure. TREX-2 is subdivided into an NPC-anchor domain (upper part) and a PCI domain part (lower part). (1) Docking to Mediator involves a conserved pair of basic Sac3 residues (red sticks) and the Med31 submodule. (2) TREX-2 regulates Cdk8 kinase module association. (3) TREX-2 impacts on RNA Pol II CTD Ser5 phosphorylation (S5; yellow). (4) TREX-2 also influences mRNA export via the same PCI surface used for interacting with Mediator. Other mRNA adaptor/export proteins are indicated. Transition between Pol II initiation and early elongation is shown. Act : transcription activator; CBC: Cap Binding Complex.

Nuclear pore complexes (NPCs) are gatekeepers at the nuclear envelope mediating traffic between the nucleus and cytoplasm. Beyond transport, NPCs dynamically interact with chromatin to regulate its architecture and activity. Genome-wide studies in yeast, flies and humans have shown that nuclear pore proteins (nucleoporins) interact with numerous active genes, but also with heterochromatin boundaries and repressed genes.^1^ The TREX-2 (Transcription-coupled Export) complex is conserved from yeast to humans and associates with the NPC via the NPC basket structure.^2,3^ The *S.cerevisiae* TREX-2 complex was found to regulate a surprisingly diverse number of chromatin-associated processes including transcription^4,5^ and mRNA export,^2,6^ targeting of activated genes to NPCs,^7^ DNA replication,^8^ and genome stability.^9^ Yeast TREX-2 is composed of Sac3, Thp1, Sem1, Sus1 and Cdc31 and can be divided into a PCI domain part (a protein scaffold also found in the Proteasome lid, CSN, and eIF3 complexes) and an NPC-basket anchor element (Fig. 1). This general domain architecture is conserved between yeast and human TREX-2.^10,11^ Like in yeast, metazoan TREX-2 plays a role in mRNA export,^12^ although its role in transcription remains to be fully explored. The molecular mechanism by which TREX-2 impacts on gene expression was a major question in the field, because of its implications for understanding how nucleoporins regulate chromatin architecture and function.

Mediator, on the other hand, is the central scaffold for transcription initiation in eukaryotes. Transcription initiation at protein-coding genes requires RNA Pol II, general transcription factors, and Mediator. Assembly of these factors on promoter DNA results in a core initiation complex, which recruits the TFIIH complex to unwind DNA and to phosphorylate the RNA Polymerase (Pol) II C-terminal domain (CTD) at Ser5. Mediator is recruited by transcription activators, stabilizes the initiation complex and stimulates TFIIH kinase activity.^13,14^

We recently reported that TREX-2 (1) interacts directly with Mediator via a specific interface of its PCI domain, (2) regulates Mediator association with the Cdk8 kinase, a central activity switch on Mediator and (3) influences RNA Pol II phosphorylation at Ser5, the defining mark of transcription initiation (Fig. 1).^15^ This has been a major advance in elucidating the biological function of TREX-2, as it uncovered a direct role of TREX-2 in modulating the core transcription machinery. TREX-2 and Mediator were shown to co-regulate a distinct subset of constitutive (e.g. the superpathway of sulfur amino acid biogenesis) and highly inducible (e.g., *GAL1* and *HXK1*) genes in yeast. Moreover, the interaction between the 2 complexes is required for the dynamic targeting of activated genes to NPCs. In essence, Mediator is a relay for communication between TREX-2 and Pol II. Here, we discuss the emerging role of promoters in regulating different aspects of mRNA metabolism and how promoter-bound Mediator and TREX-2 could impact on these transactions.

### RNA Pol II-dependent coupling events during early transcription

The biogenesis of eukaryotic mRNAs involves not only pre-mRNA synthesis by RNA Pol II, but also cotranscriptional RNA processing comprising 5′ capping, intron splicing, 3′ RNA cleavage and polyadenylation (3′ processing). The mature mRNA is packaged with RNA-binding proteins into messenger ribonucleoprotein particles (mRNPs), which is essential for its export to the cytoplasm and protein synthesis.^16,17^ The prevailing view is that, in contrast to a simple linear assembly line, gene expression machines are extensively coupled to ensure that each step in gene expression is timely, accurate and efficient. Coupling is a term that generally refers to the enhancement of one step in gene expression by another and can reflect physical and/or functional interactions between factors. This topic has been the subject of several excellent reviews.^18-20^ Here, we will focus on Pol II-dependent coupling events that accompany the earliest steps of transcription.

A prime example for sequential coupling reactions is the RNA Pol II C-terminal domain (CTD), which undergoes dynamic phosphorylation as the Pol II progresses through initiation, elongation, and termination.^21^ CTD phosphorylation creates sequential “landing pads” for numerous mRNP components.^18^ The first mark to appear on the Pol II CTD is the phosphorylation of Ser5, which is catalyzed by yeast Kin28 (human CDK7), a component of TFIIH. Mediator stimulates Ser5 CTD phosphorylation,^22^ which is thought to promote the eviction of Pol II from the promoter-bound preinitiation complex and transition to elongation.^23-25^ Beyond promoting Pol II promoter escape, Ser5 phosphorylation is linked to the capping of the pre-mRNA transcript, the very first event in co-transcriptional RNA processing.^26^ Recruitment of the capping enzyme (yeast Cet1/Ceg1) involves binding of Ceg1 to the Ser5 phosphorylated CTD of Pol II, which occurs during transcription initiation. Notably, the capping enzyme docks onto the Pol II wall in immediate proximity of the mRNA exit tunnel of Pol II. mRNA has to be at least 15 nucleotides in length to reach the Pol II surface where capping can occur. Thus, the first steps in pre-mRNA capping take place when the nascent RNA reaches the polymerase surface. The physical coupling of capping enzymes with RNA Pol II nicely illustrates how cells achieve seamless protection of RNA from degradation by 5′-exonucleases right from the start of transcription.

A common model is that acquisition of export competence begins with the recruitment of the conserved TREX (Transcription-coupled Export) complex.^16,17^ TREX also functions in various aspects of co-transcriptional mRNP formation, yet, exhibits no protein homology with TREX-2. In yeast, TREX is continuously loaded onto emerging transcripts during transcription elongation, which facilitates folding of nascent transcripts into mRNPs and helps to recruit additional RNA-binding proteins.^27-29^ When exactly the first mRNA export adaptor binds to the nascent mRNA is not well established in yeast, but intriguing insights came from studies in *Xenopus* already in 1990, which showed that the 5′ cap and the interacting cap binding complex (CBC20/CBC80 in metazoa) are required for mRNA export.^30^ Furthermore, the human TREX complex is recruited in a cap- and splicing-dependent manner to the 5′ end of the mRNA. This recruitment requires the cap binding subunit CBP80, which interacts directly with human TREX.^31^ These observations highlight the fact that the earliest steps of transcription are characterized by several interconnected events with the potential to imprint the destination of the transcript.

Our study revealed an unexpected link between TREX-2 and Mediator and the phosphorylation status of RNA Pol II. Specifically, we found that a loss of TREX-2 function leads to an increase of Ser5 phosphorylation of the Pol II CTD, which indicated that TREX-2 may have a regulatory impact when bound to Mediator. In support of this notion, we found that TREX-2 binds directly to the Med31/Med7 submodule, which is positioned in proximity to the Pol II CTD binding site on Mediator. Whether and how the impact of TREX-2 on the Pol II “phospho-code” plays a role in choreographing subsequent mRNA processing events will require further analysis.

### Role of promoters in mRNA metabolism

Since TREX-2 is necessary for mRNA export, its interaction with promoter-bound Mediator raises the interesting question whether the mRNA export function of TREX-2 is linked to Mediator's role in initiating transcription. This would imply that cells connect the earliest and latest steps of nuclear gene expression. Notably, a growing number of studies has reported promoter-dependent effects on downstream mRNA metabolism, which altogether raise the question whether transcription initiation is indeed a promoter's only task. In 2011, 2 studies showed that transcription factors and DNA promoters can directly influence the stability of the transcripts that they produce, independent of the transcript sequence. One paper described destabilization of *SWI5* and *CLB2* mRNAs at the onset of metaphase in *S.cerevisiae*.^32^ This stability switch requires promoter-dependent deposition of the mitotic exit kinase Dbf2 on both mRNAs during transcription. The second paper described a similar decay-enhancing effect mediated by the transcription factor Rap1.^33^ Rap1 stimulates both the synthesis and the decay of a specific population of endogenous mRNAs suggesting that Rap1 association with the promoter affects the composition of the exported mRNP, which in turn regulates mRNA decay in the cytoplasm. More recently, yeast promoter sequences were shown to direct both the localization of mRNAs and their translation during starvation.^34^ *S.cerevisiae* responds to glucose starvation by translating a subset of transcriptionally activated mRNAs while decreasing translation of others. The information specifying the differential localization and protein production of these 2 classes of mRNAs was found to depend on specific promoters targeted by heat-shock factor 1 (Hsf1). Last but not least, promoter-proximal pausing was shown to be involved in regulating alternative cleavage and polyadenylation of mRNAs in *Drosophila* neurons.^35^ In flies, the RNA-binding protein ELAV (Embryonic Lethal Abnormal Visual System) inhibits RNA processing at proximal polyadenylation sites, thereby promoting the formation of exceptionally long 3′UTRs. Paused Pol II promotes recruitment of ELAV and leads to extended genes, and this effect is regulated by the promoter. In sum, promoters can influence gene expression by mechanisms other than transcriptional control, perhaps through mediating the loading of proteins onto mRNAs.

The emerging theme of all these studies is an impact of promoters on gene expression that clearly goes beyond transcription initiation. Promoters appear to reach out to influence later steps of mRNA processing and mRNP formation. A central assumption underlying promoter-coupled mRNA processing events is that promoter elements are somehow brought into physical proximity with the nascent mRNA to allow several types of molecular cross-talk. However, the molecular details of these transactions are largely unclear.

### Mediator as an emerging regulator of mRNA processing

Given the influence of promoter-bound Mediator on Pol II Ser5 phosphorylation and the role of the CTD in scaffolding various mRNA processing and packaging events, it is conceivable that Mediator impacts on the mRNA life cycle through setting the proper RNA Pol II “phospho code.” But is there any evidence for a direct role of Mediator in dictating mRNA fate? Surprising insights came from a study, which reported direct physical interactions between the Mediator subunit MED23 and the hnRNP L protein, a regulator of alternative splicing in metazoans.^36^ Functionally, MED23 regulates a subset of alternative splicing and alternative cleavage and polyadenylation events. These results suggested a crosstalk between Mediator and the splicing machinery and advanced the idea that Mediator could be involved in coupling transcription initiation with mRNA processing possibly by serving as a stepping-stone for splicing-related factors. Although yeast has no MED23 ortholog, the role of Mediator in scaffolding diverse promoter-functions could be a conserved property. We propose that Mediator's interaction with TREX-2 could similarly mediate the coupling of transcription initiation and export of specific mRNAs through the nuclear pore.

### The role of TREX-2 in transcription and mRNA export

A surprising finding of our study was that TREX-2 employs identical Sac3 PCI domain residues to promote both transcription and mRNA export. TREX-2 directly interacts with the Mediator Med31/Med7 submodule via conserved polar residues on the Sac3 PCI domain surface. Notably, TREX-2 uses the same polar residues to contact Mediator and to regulate mRNA export. This could indicate that TREX-2 is involved in a sequential coupling reaction, in which binding to Med31/Med7 is displaced by an interaction with a yet unknown mRNP factor. TREX-2 could therefore either promote the loading of factors onto the nascent mRNA or become itself loaded onto RNA. These possibilities remain to be explored, but are conceivable given that mutually exclusive protein-protein and protein-RNA interactions are a common feature of many co-transcriptional events. Such handover reactions are thought to impose directionality and a sequential order to mRNP formation.^20^

TREX-2 also binds to the general mRNA exporter Mex67/Mtr2 ^2^ as well as DNA or RNA *in vitro*.^11^ Moreover, TREX-2 was suggested to undergo large-scale conformational changes from extended to more compact forms *in vitro*.^37^ The functional relevance of this structural plasticity and the spatiotemporal order of TREX-2 interactions with Mediator, Mex67/Mtr2 and the NPC basket during gene expression will have to be explored in detail. Such studies will be essential to understand the precise mechanism by which TREX-2 promotes the coupling of transcription and mRNA export in cells.

## Conclusion and perspective

We have recently described a direct link between the TREX-2 complex and Mediator using structural biology, *in vitro* biochemical reconstitution and various *in vivo* functional analyses. These findings are key to understanding how NPC-associated adaptors modulate transcription, mRNA export and nuclear gene localization. This has revealed a molecular mechanism of TREX-2 function and opens new avenues for exploring how promoters can regulate multiple aspects of mRNA biology beyond transcription initiation.
